# Relationships among Trait EI, Need Fulfilment, and Performance Strategies

**DOI:** 10.3390/jfmk4030050

**Published:** 2019-07-31

**Authors:** Nadia Barberis, Danilo Calaresi, Maria Cristina Gugliandolo

**Affiliations:** 1 Department of Clinical and Experimental Medicine, University of Messina, 98122 Messina, Italy; 2 Department of Human, Social and Health Sciences, University of Cassino and South Latium, 03100 Cassino, Italy

**Keywords:** self-determination theory, sport, emotional intelligence

## Abstract

Performance strategies used in sport have been the focus of many sport enhancement interventions, and are considered important factors for describing behavior in sport. Several studies have shown that both trait emotion intelligence (trait EI) and satisfaction of basic needs in sport are relevant aspects of performance strategies used by athletes; however, it seems these two aspects were never tested concurrently, in an integrated framework. The aim of this study was to test a mediational model of psychological basic needs in the relationship between trait EI and performance strategies in sports. In a sample of 187 participants, aged between 16 and 25 years old (M_age_ = 20.55; SD = 3.39), instruments were administered to measure trait EI, satisfaction of basic needs, and performance strategies in sport. Results of this study showed that trait EI was related to performance strategies in sport and to satisfaction of basic needs, as well as that satisfaction of basic needs was related to performance strategies in sport. Furthermore, satisfaction of basic needs has shown a mediational role in the relation between trait EI and performance strategies in sport.

## 1. Introduction

In recent years, many sport psychologists have been focused on exploring which psychological skills may have a relevant impact on the performance strategies adopted by athletes to achieve success in sports [1,2]. For example, there has been a growing interest in the role played by the emotions in athletes [3] and the relationship between the satisfaction of basic needs and sports [4]. Many sport enhancement interventions, in fact, focus their attention on the empowerment of such psychological aspects, as these are considered important factors for predicting behavior and performance in sport [5].

### 1.1. Performance Strategies in Sport

In recent decades, the conceptualization and measurement of athletes’ psychological strategies has been of great interest to sport psychologists [6]. 

Several studies have been focused on assessing the psychological strategies deployed by athletes, in consideration of their close link with performance in sport during both competition and training [7]. Surely, psychological assessment is a fundamental prerequisite for any sport psychology intervention [8]. 

Individual peculiarities at the psychological level, making each athlete different, are definitely important for describing and predicting behavior and performance [9]. Indeed, behavioral change skills and strategies form the focus of many athletic interventions, which aim to empower the performance of the athletes [10].

### 1.2. Trait Emotional Intelligence and Performance Strategies in Sport

In recent years, there has been a growing interest regarding the effect of emotions on athletic performance [3]. In particular, several studies have underlined a significant relationship between trait emotional intelligence (EI) and sport performance [1]. 

Research shows that emotional intelligence and emotional states are predictive of sports performance [11], and that athletes manage emotions correspondingly [12]. Emotionally intelligent athletes are able to get themselves into the appropriate emotional states for the needs of the context, so, if the context requires high stimulation, emotionally intelligent people are skilled in preparing themselves mentally [13]. People constantly track their emotional states and develop self-regulation skills to preserve or modify their emotions to reasonable levels; in fact, if there is some degree of dissimilarity between an individual’s present and desired feelings, then regulatory processes are activated [14].

Trait EI (or trait emotional self-efficacy) refers to a set of personal perceptions of one’s emotional abilities, and should be assessed using rating scales and questionnaires [15]. In contrast, ability EI is conceptualized as a cognitive ability necessary to grasp and regulate emotions and should be assessed through tests of maximum performance [16].

### 1.3. Satisfaction of Basic Needs and Performance Strategies in Sport

The Self Determination Theory (SDT) [17] is a meta-theory according to which an individual is an active organism that aspires to fully develop his abilities and to satisfy his satisfaction of basic needs [17]. 

Satisfaction of basic needs theory (BPNT) [18], a subtheory within SDT, proposes that people function and develop most effectively as a consequence of social–environmental support for their autonomy, competence, and relatedness needs. The need to feel autonomous concerns psychological freedom and the experience of one’s own will. The need to be competent refers to the adequacy of one’s abilities in pursuing goals or performing specific tasks. The need to relate to other subjects concerns the field of treatment and mutual concern with respect to other subjects [19].

Several studies concerning several fields, such as parenting, work, and sport, have underlined a strong association between psychological need satisfaction (or frustration) and well-being [20,21,22]. As far as sport psychology is concerned, research has explored basic psychological needs with regard to several constructs linked to performance strategies in sport, such as motivation [2] and athlete engagement [4]. Moreover, research has also shown that the satisfaction of basic needs can enhance athletes’ task orientation and overall sport performance [23].

A construct associated with need satisfaction/frustration is that of need thwarting, which is conceptualized as “the perception that need satisfactions are being obstructed or actively frustrated within a given context” [24]. Unlike “need frustration”, great emphasis is placed on the perception aspect of the construct, on the active part the context has in frustrating such needs, and on the feelings of oppression and inadequacy an individual experiences when his/her psychological needs are thwarted [24]. It seems that need thwarting is far more likely to lead to negative outcomes, compared to need frustration [25], even in contexts such as sport [24].

### 1.4. The Present Study

The above studies have underlined that both trait EI and satisfaction of basic needs might be influential aspects of performance strategies in sport; however, these two aspects have not been tested concurrently, to our knowledge. 

Trait EI is a construct that includes several emotion-related self-perceptions that research has linked to need fulfilment, such as empathy, self-motivation, self-esteem, assertiveness, and stress management [26]. Several studies have underlined that need fulfilment predicts happiness, self-esteem, and self-motivation [27]. Ahmad, Vansteenkiste, and Soenens [28] have shown that need fulfilment is linked to several variables, such as task orientation, peer sociability, and assertiveness. Individuals who experience high levels of need fulfilment in their lives may also be likely to demonstrate high levels of emotional intelligence because they can decide to express what they feel (autonomy), find skillful ways to control their emotions (competence), and show affection to those close to them and ask for emotional support (relatedness) [26].

A difficulty in managing emotional skills, linked to frustrated or thwarted satisfaction of basic needs, could imply the activation of compensatory processes like suboptimal performance strategies in sport. At the same time, satisfying levels of need fulfillment could help to prevent the application of suboptimal performance strategies in sport by finding ways to express and control emotions, and by asking for emotional support.

For these reasons, the aim of this study was to verify the relationships between trait EI, need fulfilment, and performance strategies in training, and the relationships between trait EI, need fulfilment, and performance strategies in competition, in athletes who compete in sports.

## 2. Materials and Methods

### 2.1. Participants

The sample consisted of a total of 187 participants, all of Italian nationality, aged between 16 and 25 years old (M = 20.55; SD = 3.39). 94 participants were females (50.3%) and 93 participants were males (49.7%). Regarding educational level, 46.5% (87) had a high school diploma, 34.8% (65) had completed secondary school, and 18.7% (35) had a degree. All participants practiced sports for a minimum of 1 h a week, up to a maximum of 30 h (M = 7.17; SD = 4.53). The sample participants were athletes of different disciplines: The majority (21.93%, *n* = 41) practiced gym activities of various kinds (weightlifting, calisthenics, yoga, body building, etc.); 20.86% (*n* = 39) were basketball players; 13.37% (*n* = 25) were soccer players; 12.84% (*n* = 24) were artistic gymnastics athletes; 8.02% (*n* = 15) practiced running as a physical activity; 5.34% (*n* = 10) were dancers; 4.27% (*n* = 8) were rhythmic gymnastics athletes; and the remaining 13.37% (*n* = 25) practiced other sports (swimming, fencing, martial arts, volleyball, artistic skating). 

### 2.2. Measures

#### 2.2.1. Trait EI

The Trait Emotional Intelligence Questionnaire—Short Form [29] is a 30-item self-report questionnaire designed to measure Trait EI. This questionnaire is a short version of the full form. The items are sampled (two items for facet) from the 15 facets of the Trait EI sampling domain (trait optimism, trait happiness, self-esteem, emotion management, assertiveness, social awareness, trait empathy, emotion perception, emotion expression, relationships, emotion regulation, impulse control, stress management, self-motivation, and adaptability). It is also possible to get a score on four factors of major relevance: Well-being (e.g., “On the whole, I’m pleased with my life”), self-control (e.g., “I usually find it difficult to regulate my emotions”), emotionality (e.g., “I’m normally able to ‘get into someone’s shoes’ and ‘experience their emotions’”), and sociability (e.g., “I would describe myself as a good negotiator”), as well as a global score. Participants are required to rate, on a 7-point scale, their level of agreement with each item. Higher scores indicate higher trait EI. The Trait Emotional Intelligence Questionnaire—SF has been widely used in several studies, showing a good level of reliability with Cronbach’s alphas that ranged from 0.67 to 0.88 and reporting a good level of construct, concurrent, and incremental validity [30].

#### 2.2.2. Needs

To assess satisfaction of the need for autonomy, five items collated by Standage, Duda, and Ntoumanis [31] were used (e.g., “I have some choice in what I want to do in my sport”). Satisfaction of the need for competence was assessed using five items (e.g., “I think I am pretty good at my sport”) from the Competence subscale of the Intrinsic Motivation Inventory (IMI) [32]. Finally, satisfaction of the need for relatedness was assessed using the 5-item Acceptance subscale of the Need for Relatedness Scale [33]. A sample item is, “When participating in my sport, I feel supported.”

Need thwarting was assessed using the 12-item Psychological Need Thwarting Scale (PNTS) [24]. The stem used in the questionnaire was, “In my sport …,” and athletes rated statements such as “I feel forced to follow training decisions made for me”, “I feel I am rejected by those around me”, and “There are times when I am told things that make me feel incompetent.” It is also possible to get a full-scale on the satisfaction and relative frustration. In line with a recent study [26], to obtain a total score for satisfaction, we took the average of the three scales relating to satisfaction of needs and the reverse score of the three scales related to the frustration of needs. Responses for all measures were provided on a 7-point scale, ranging from 1 (strongly disagree) to 7 (strongly agree). The subscales have demonstrated satisfactory levels of internal reliability in previous research conducted in the sport domain [34].

#### 2.2.3. Performance Strategies

The Test of Performance Strategies (TOPS) [35] is a 64-item self-report questionnaire designed to measure the psychological skills and strategies used by athletes in competition (32 items) and during practice (32 items). There are nine factors assessed by the instrument: Attentional Control/Negative Thinking, Goal Setting, Imagery, Relaxation, Activation, Self-Talk, Emotional Control, Automaticity. The first group of items (competition) does not assess “Attentional Control”. The second group of items (practice) does not assess “Negative Thinking”. The remaining seven factors are shared between the two groups of items. Participants are required to state, on a 5-point scale, the frequency with which they deploy each of the psychological strategies included in the test. The Test of Performance Strategies is currently considered to be one of the most widely used tests in the field of sports psychology and research has provided evidence of the construct validity and internal consistency of the TOPS subscales [6].

### 2.3. Procedures

The participants were recruited in two different ways. Half of the participants were recruited through mails, with the collaboration of the Regional Committee FGI Calabria, and through social networks. The other half were recruited at gyms and sport centers. Administration took place in a calm and peaceful environment in the presence of a psychology student, and all participants signed an informed consent form before taking the test. All were guaranteed the anonymity of their data. The protocol took about 30 min to be completed. The data were then analyzed using IBM SPSS-22 and AMOS.

### 2.4. Data Analyses

The descriptive analyses and correlations were analyzed using SPSS-22. The first and second model were tested using AMOS, with the Maximum Likelihood Method. The third and fourth model were tested using AMOS, with the Maximum Likelihood Method. Additionally, in line with the recommendations outlined by Preacher and Hayes [36], confidence intervals of the direct, indirect, and total effects with 5000 bootstrap replication samples based on the original sample were used [37].

## 3. Results

### 3.1. Descriptive and Correlations

Scale reliabilities, means, standard deviations, skewness, and kurtosis of the scores of each variable are shown in Table 1 and Table 2. Furthermore, Table 1 and Table 2 show the correlations among the dimensions of the questionnaires.

Regarding the training strategies, Trait EI positively correlated with Goal Setting, Emotional Control, Activation, Self-Talk, and Attentional Control. Need Fulfilment positively correlated with Goal Setting, Automaticity, Emotional Control, Activation, Self-Talk, and Attentional Control.

Regarding the competition strategies, Trait EI positively correlated with Emotional Control, Imagery, Activation, and Relaxation, and negatively correlated with Automaticity and Negative Thinking. Need Fulfilment positively correlated with Goal Setting, Emotional Control, Imagery, Activation, Self-Talk, and Relaxation and negatively correlated with Automaticity and Negative Thinking. Moreover, Need Fulfilment positively correlated with Trait EI.

### 3.2. Mediation Model

**Figure 1:**
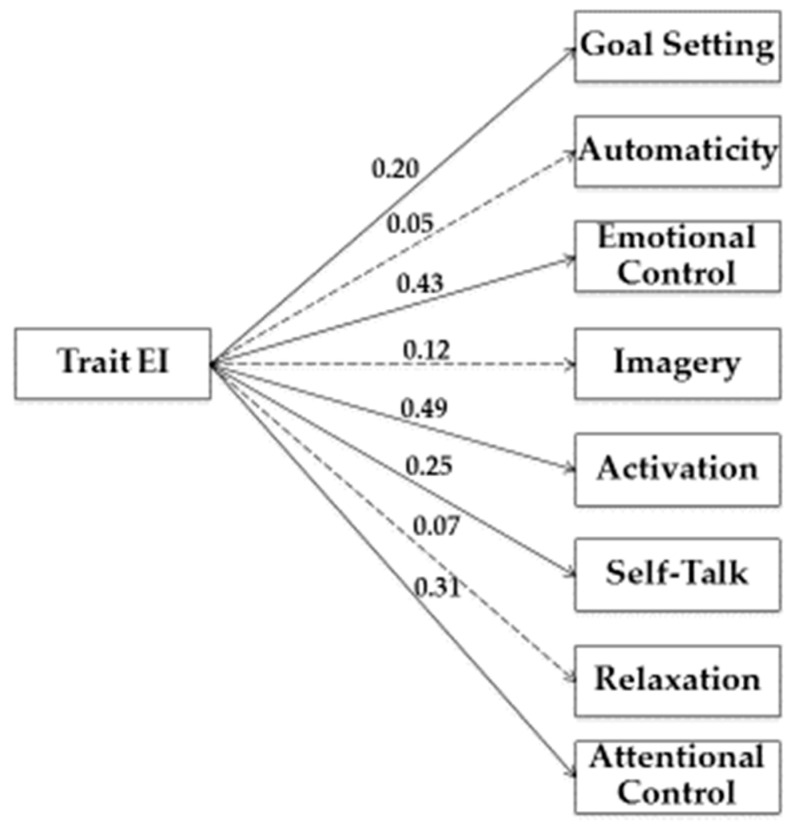
Structural model of associations between Trait EI, Need Fulfiment, and training strategies. Note: Coefficients shown are standardized path coefficients. Dotted lines represent non-significant relations.

**Figure 2:**
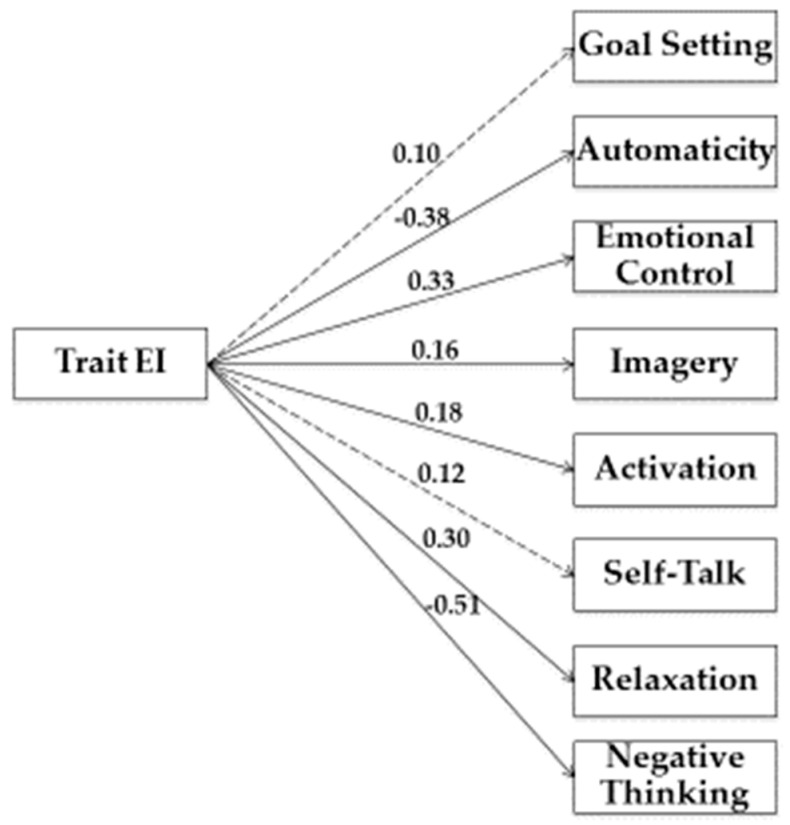
Structural model of associations between Trait EI, Need Fulfiment, and competition strategies. Note: Coefficients shown are standardized path coefficients. Dotted lines represent non-significant relations.

**Figure 3:**
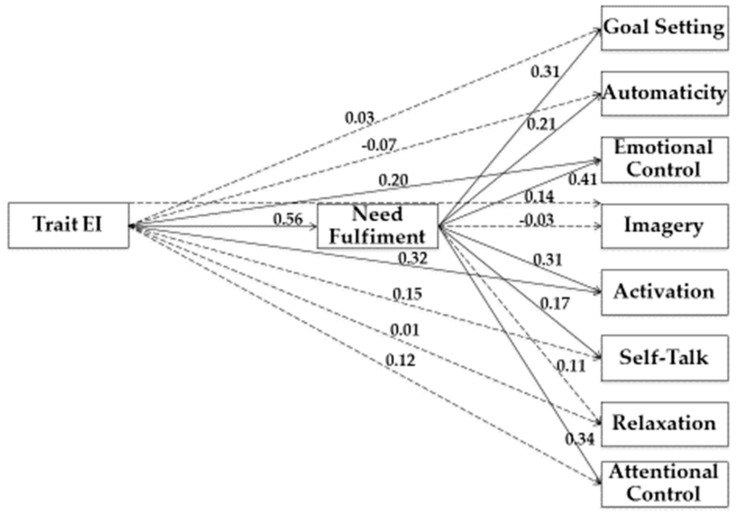
Structural model of associations between Trait EI, Need Fulfiment, and training strategies. Note: Coefficients shown are standardized path coefficients. Dotted lines represent non-significant relations.

**Figure 4:**
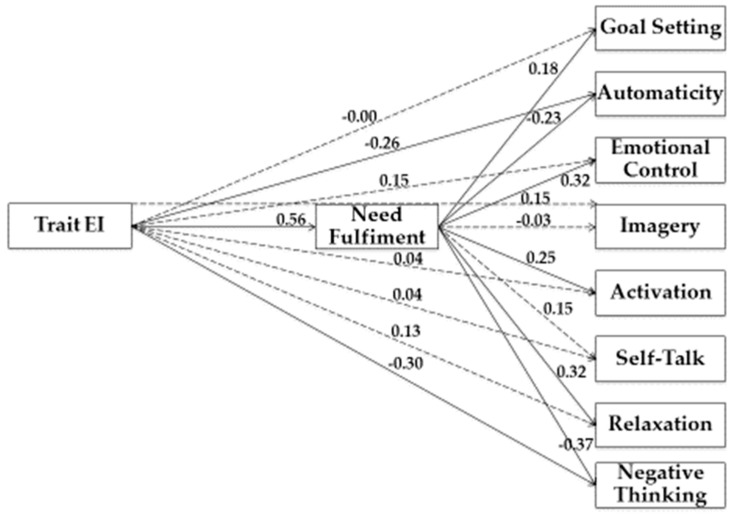
Structural model of associations between Trait EI, Need Fulfiment, and competition strategies. Note: Coefficients shown are standardized path coefficients. Dotted lines represent non-significant relations.

In our study, we used Structural Equation Modeling (SEM) to examine the relationship between variables. As shown in Figure 1, Figure 2, Figure 3 and Figure 4, we tested four models.

In the first model, (see Figure 1) Trait EI was the predictor variable, while Goal Setting, Automaticity, Emotional Control, Imagery, Activation, Self-Talk, Relaxation, and Attentional Control represented criterion variables.

Estimation of the saturated model (no fit indices were reported) showed a significant path from trait EI to Goal Setting (β = 0.20; *p* < 0.05), Emotional Control (β = 0.43; *p* < 0.05), Activation (β = 0.49; *p* < 0.05), Self-Talk (β = 0.25; *p* < 0.05), and Attentional Control (β = 0.31; *p* < 0.05).

In the second model, (see Figure 2) Trait EI was the predictor variable, while Goal Setting, Automaticity, Emotional Control, Imagery, Activation, Self-Talk, Relaxation, and Negative Thinking represented criterion variables.

Estimation of the saturated model (no fit indices were reported) showed a significant path from trait EI to Automaticity (β = −0.38; *p* < 0.05), Emotional Control (β = 0.33; *p* < 0.05), Imagery (β = 0.16; *p* < 0.05), Activation (β = 0.18; *p* < 0.05), Relaxation (β = 0.30; *p* < 0.05), and Negative Thinking (β = −0.51; *p* < 0.05).

In the third model, (see Figure 3) Trait EI was the predictor variable, Need Fulfilment was the mediator variable, while Goal Setting, Automaticity, Emotional Control, Imagery, Activation, Self-Talk, Relaxation, and Attentional Control represented criterion variables. Analysis of the covariance matrices was tested using AMOS 18 and solutions were generated based on maximum likelihood estimation.

To explore the significance of the indirect effects that emerged (i.e., drop from total to direct effect), we used the bootstrap-generated bias-corrected confidence interval approach [36]. Estimation of the saturated model (no fit indices were reported), as shown in Table 3, underlined a statistically significant indirect association between Trait EI and Goal Setting (β = 0.17; *p* < 0.001), Automaticity (β = 0.12; *p* < 0.01), Emotional Control (β = 0.23; *p* < 0.001), Activation (β = 0.17; *p* < 0.001), Self-Talk (β = 0.09; *p* = 0.04), and Attentional Control (β = 0.19; *p* < 0.001), through Need.

Furthermore, there was a statistically significant direct association between Trait EI and Need (β = 0.56; *p* < 0.001), Emotional Control (β = 0.20; *p* < 0.01), and Activation (β = 0.32; *p* < 0.001). Moreover, there was a statistically significant direct association between Need and Goal Setting (β = 0.31; *p* < 0.001), Automaticity (β = 0.21; *p* < 0.01), Emotional Control (β = 0.41; *p* < 0.001), Activation (β = 0.31; *p* < 0.001), Self-Talk (β = 0.17; *p* = 0.04), and Attentional Control (β = 0.34; *p* < 0.001).

In the fourth model (see Figure 4), Trait EI was the predictor variable and Need Fulfilment was the mediator variable, while Goal Setting, Automaticity, Emotional Control, Imagery, Activation, Self-Talk, Relaxation, and Negative Thinking represented criterion variables. Analysis of the covariance matrices was conducted using EQS 6.2 and solutions were generated based on maximum likelihood estimation.

To explore the significance of the indirect effects that emerged (i.e., drop from total to direct effect), we used the bootstrap-generated bias-corrected confidence interval approach [36]. Estimation of the saturated model (no fit indices were reported), as shown in Table 4, underlined a statistically significant indirect association between Trait EI and Goal Setting (β = 0.10; *p* < 0.001), Automaticity (β = −0.13; *p* < 0.001), Emotional Control (β = 0.18; *p* < 0.001), Imagery (β = 0.08; *p* = 0.03), Activation (β = 0.14; *p* < 0.001), Relaxation (β = 0.18; *p* < 0.001), Negative Thinking (β = −0.21; *p* < 0.001), through Need.

Furthermore, there was a statistically significant direct association between Trait EI and Need (β = 0.56; *p* < 0.001), Automaticity (β = −0.26; *p* < 0.01), and Negative Thinking (β = −0.30; *p* < 0.001). Moreover, there was a statistically significant direct association between Need and Goal Setting (β = 0.18; *p* = 0.04), Automaticity (β = −0.23; *p* = 0.02), Emotional Control (β = 0.32; *p* < 0.001), Activation (β = 0.25; *p* < 0.01), Relaxation (β = 0.32; *p* < 0.001), and Negative Thinking (β = −0.37; *p* < 0.001).

## 4. Discussion

The purpose of this study was to test a mediational model in which the relationship between trait EI and performance strategies in sport was mediated by need fulfilment.

The results of this study, in line with the findings of this research field [1,22], showed that trait EI was positively related to performance strategies in sport, both in training and competition contexts, and positively related to need fulfilment, whereas need fulfillment was positively related to performance strategies in sport, both in training and competition contexts. Furthermore, need fulfilment showed a mediation role in the relationship between trait EI and performance strategies in sport, both in training and competition contexts.

As expected, both trait EI and need fulfilment were positively associated with optimal performance strategies in sport. Need satisfaction involves the capability to express and control emotions and to ask for emotional support, which is related to several key factors in sport, such as motivation [2] and athlete engagement [4]. At the same time, high levels of emotional intelligence involve the capability of regulating emotions, which seem to be predictive of sport performance [11]; in particular, if it is also considered that athletes manage emotions accordingly to feedback received by the sport environment [12].

This study provides a relevant contribution to the relationship between trait EI and need fulfilment in sports. High levels of emotional intelligence, by having the tools to manage emotions accordingly [12], may allow the satisfaction of basic needs, by promoting ways to express and control emotions and to ask for emotional support [19], which in turn might also enable the use of optimal performance strategies in sport.

For these reasons, performance strategies in sport may not only be a consequence of high emotional intelligence, but also a result of need satisfaction, which accompanies these emotional states. The results seem to suggest that trait EI is a plausible antecedent of performance strategies in sport, accounting for individual variations in need fulfilment in sport, which results in different levels of psychological well-being in athletes [22], and of participation and success in sport [2,4].

In the present study, there are some limitations to take into consideration. First of all, it should be highlighted that no power analysis was carried out to estimate the number of participants to recruit. Secondly, in this study, we used a convenience sample that involved psychology students and friends and this could have created bias in the research, considering that the sample might have characteristics that do not necessarily represent the general population. Moreover, the exclusive use of self-reports could have increased measurement bias, so future research could try to use objective measures and/or multi-rating sources. It should also be underlined that the cross-sectional design of the study prevented the drawing of causal inferences. Experimental design is recommended for future research.

Despite the above limitations, this study provides relevant theoretical and practical insights. Firstly, from a theoretical point of view, integrating aspects of SDT and trait EI theory should provide a more coherent and exhaustive model for the understanding of psychological processes. Secondly, from a practical point of view, the results underline the fact that athletes with fulfilled satisfaction of basic needs and high trait EI make use of optimal performance strategies in sport. For this reason, training programs in sport psychology could emphasize the promotion of psychological needs fulfilment and trait EI to increase their effectiveness.

## Figures and Tables

**Table 1:** Descriptive Analysis and Correlations in training contexts.

		α	M	SD	Skew	Kurt	1	2	3	4	5	6	7	8	9
1	Trait EI	0.90	4.73	0.68	−0.09	−0.40									
2	Needs Fulfilment	0.93	5.14	0.93	−0.19	−0.44	0.56 **								
3	Goal Setting in training	0.73	3.78	0.74	−0.36	−0.31	0.20 **	0.32 **							
4	Imagery in training	0.61	3.21	0.83	0.19	−0.40	0.12	0.05	0.45 **						
5	Relaxation in training	0.77	2.50	0.89	0.21	−0.78	0.07	0.12	0.16 *	0.20 **					
6	Activation in training	0.66	3.56	0.73	0.03	−0.43	0.49 **	0.49 **	0.49 **	0.29 **	0.15 *				
7	Self-Talk in training	0.82	3.43	0.95	−0.36	−0.49	0.25 **	0.25 **	0.52 **	0.44 **	0.37 **	0.45 **			
8	Emotional control in training	0.65	3.18	0.80	−0.05	−0.63	0.43 **	0.52 **	0.03	0.06	0.15 *	0.38 **	0.30 **		
9	Automaticity in training	0.62	3.10	0.57	−0.02	−0.16	0.05	0.17 *	0.20 **	0.10	0.26 **	0.22 **	0.34 **	0.15 *	
10	Attentional Control in training	0.69	3.55	0.79	−0.21	−0.59	0.31 **	0.40 **	0.45 **	0.28 **	0.05	0.56 **	0.31 **	0.32 **	0.08

Note: N = 187; * *p* < 0.01; ** *p* < 0.05.

**Table 2:** Descriptive Analysis and Correlations in competition contexts.

		α	M	SD	Skew	Kurt	1	2	3	4	5	6	7	8	9
1	Trait EI	0.90	4.73	0.68	−0.09	−0.40									
2	Needs Fulfilment	0.93	5.14	0.93	−0.19	−0.44	0.56 **								
3	Goal Setting in competition	0.79	3.66	0.84	−0.16	−0.78	0.10	0.18 *							
4	Imagery in competition	0.76	3.39	0.87	−0.08	−0.54	0.16 *	0.19 **	0.54 **						
5	Relaxation in competition	0.79	3.02	0.87	−0.15	−0.27	0.30 **	0.39 **	0.14 *	0.40 **					
6	Activation in competition	0.75	3.64	0.74	−0.07	−0.74	0.18 *	0.27 **	0.54 **	0.57 **	0.45 **				
7	Self-Talk in competition	0.83	3.37	0.96	−0.38	−0.43	0.12	0.17 *	0.44 **	0.54 **	0.36 **	0.64 **			
8	Emotional control in competition	0.62	3.31	0.84	−0.25	−0.44	0.33 **	0.41 **	0.03	0.18	0.55 **	0.24 **	0.14		
9	Automaticity in competition	0.60	2.36	0.69	0.20	−0.53	−0.38	−0.37 **	−0.19 *	−0.26 **	−0.26	−0.27 **	−0.17 *	−0.42 **	
10	Negative Thinking in competition	0.72	2.34	0.82	0.43	−0.47	−0.51 **	−0.54 **	−0.13	−0.24 **	−0.51 **	−0.32 **	−0.27 **	−0.58 **	0.37 **

Note: N = 187; * *p* < 0.01; ** *p* < 0.05.

**Table 3:** Direct Effects, Indirect Effects, and Total Effects in training contexts.

	β	Lower Bound (BC) 95% CI	Upper Bound (BC) 95% CI
*Direct Effects*			
Trait EI → Need Fulfilment	0.56	0.45	0.65
Trait EI → Goal Set	0.03	−0.13	0.19
Trait EI → Automaticity	−0.07	−0.23	0.11
Trait EI → Emotional Control	0.20	0.05	0.33
Trait EI → Imagery	0.14	−0.04	0.30
Trait EI → Activation	0.32	0.16	0.47
Trait EI → Self-Talk	0.15	−0.01	0.30
Trait EI → Relaxation	0.01	−0.16	0.18
Trait EI → Attentional Control	0.12	−0.04	0.27
Need Fulfilment → Goal Setting	0.31	0.14	0.46
Need Fulfilment → Automaticity	0.21	0.06	0.36
Need Fulfilment → Emotional Control	0.41	0.27	0.55
Need Fulfilment → Imagery	−0.03	−0.19	0.14
Need Fulfilment → Activation	0.31	0.15	0.47
Need Fulfilment → Self-Talk	0.17	0.01	0.32
Need Fulfilment → Relaxation	0.11	−0.09	0.32
Need Fulfilment → Attentional Control	0.34	0.19	0.47
*Indirect Effects*			
Trait EI → Need Fulfilment → Goal Setting	0.17	0.08	0.27
Trait EI → Need Fulfilment → Automaticity	0.12	0.04	0.21
Trait EI → Need Fulfilment → Emotional Control	0.23	0.15	0.33
Trait EI → Need Fulfilment → Imagery	−0.02	−0.11	0.08
Trait EI → Need Fulfilment → Activation	0.17	0.08	0.28
Trait EI → Need Fulfilment → Self-Talk	0.09	0.01	0.19
Trait EI → Need Fulfilment → Relaxation	0.06	−0.05	0.18
Trait EI → Need Fulfilment → Attentional Control	0.19	0.11	0.28
*Total Effects*			
Trait EI → Goal Setting	0.20	0.06	0.33
Trait EI → Automaticity	0.05	−0.10	0.21
Trait EI → Emotional Control	0.43	0.31	0.52
Trait EI → Imagery	0.12	−0.02	0.26
Trait EI → Activation	0.49	0.37	0.60
Trait EI → Self-Talk	0.25	0.10	0.38
Trait EI → Relaxation	0.07	−0.07	0.20
Trait EI → Attentional Control	0.31	0.18	0.43
Need Fulfilment → Goal Setting	0.31	0.14	0.46
Need Fulfilment → Automaticity	0.21	0.06	0.36
Need Fulfilment → Emotional Control	0.41	0.27	0.55
Need Fulfilment → Imagery	−0.03	−0.19	0.14
Need Fulfilment → Activation	0.31	0.15	0.47
Need Fulfilment → Self-Talk	0.17	0.01	0.32
Need Fulfilment → Relaxation	0.11	−0.09	0.32
Need Fulfilment → Attentional Control	0.34	0.19	0.47

Note: BC 95% CI = Bias-Corrected Confidence Interval.

**Table 4:** Direct Effects, Indirect Effects, and Total Effects in competition contexts.

	β	Lower Bound (BC) 95% CI	Upper Bound (BC) 95% CI
*Direct Effects*			
Trait EI → Need Fulfilment	0.56	0.45	0.65
Trait EI → Goal Set	−0.00	−0.16	0.16
Trait EI → Automaticity	−0.26	−0.43	−0.07
Trait EI → Emotional Control	0.15	−0.01	0.31
Trait EI → Imagery	0.07	−0.10	0.23
Trait EI → Activation	0.04	−0.13	0.21
Trait EI → Self-Talk	0.04	−0.12	0.20
Trait EI → Relaxation	0.13	−0.05	0.30
Trait EI → Negative Thinking	−0.30	−0.44	−0.16
Need Fulfilment → Goal Setting	0.18	0.01	0.34
Need Fulfilment → Automaticity	−0.23	−0.40	−0.05
Need Fulfilment → Emotional Control	0.32	0.18	0.46
Need Fulfilment → Imagery	0.15	−0.00	0.29
Need Fulfilment → Activation	0.25	0.09	0.40
Need Fulfilment → Self-Talk	0.15	0.01	0.30
Need Fulfilment → Relaxation	0.32	0.15	0.47
Need Fulfilment → Negative Thinking	−0.37	−0.50	−0.24
*Indirect Effects*			
Trait EI → Need Fulfilment → Goal Setting	0.10	0.01	0.20
Trait EI → Need Fulfilment → Automaticity	−0.13	−0.23	−0.03
Trait EI → Need Fulfilment → Emotional Control	0.18	0.10	0.27
Trait EI → Need Fulfilment → Imagery	0.09	0.00	0.17
Trait EI → Need Fulfilment → Activation	0.14	0.05	0.24
Trait EI → Need Fulfilment → Self-Talk	0.08	−0.01	0.17
Trait EI → Need Fulfilment → Relaxation	0.18	0.09	0.27
Trait EI → Need Fulfilment → Negative Thinking	−0.21	−0.30	−0.13
*Total Effects*			
Trait EI → Goal Setting	0.10	−0.04	0.24
Trait EI → Automaticity	−0.38	−0.50	−0.25
Trait EI → Emotional Control	0.33	0.20	0.45
Trait EI → Imagery	0.16	0.01	0.30
Trait EI → Activation	0.18	0.03	0.32
Trait EI → Self-Talk	0.12	−0.03	0.26
Trait EI → Relaxation	0.30	0.16	0.43
Trait EI → Negative Thinking	−0.51	−0.60	−0.40
Need Fulfilment → Goal Setting	0.18	0.01	0.34
Need Fulfilment → Automaticity	−0.23	−0.40	−0.05
Need Fulfilment → Emotional Control	0.32	0.18	0.46
Need Fulfilment → Imagery	0.15	−0.00	0.29
Need Fulfilment → Activation	0.25	0.09	0.40
Need Fulfilment → Self-Talk	0.15	−0.01	0.30
Need Fulfilment → Relaxation	0.32	0.15	0.47
Need Fulfilment → Negative Thinking	−0.37	−0.50	−0.24

Note: BC 95% CI = Bias-Corrected Confidence Interval.
